# Changes and prognostic impact of inflammatory nutritional factors during neoadjuvant chemoradiotherapy for patients with resectable and borderline resectable pancreatic cancer

**DOI:** 10.1186/s12876-020-01566-8

**Published:** 2020-12-14

**Authors:** Minoru Oshima, Keiichi Okano, Hironobu Suto, Yasuhisa Ando, Hideki Kamada, Tsutomu Masaki, Shigeo Takahashi, Toru Shibata, Yasuyuki Suzuki

**Affiliations:** 1 Department of Gastroenterological Surgery, Faculty of Medicine, Kagawa University, 1750-1, Ikenobe, Miki-cho, Kita-gun, Kagawa 761-0793 Japan; 2 Departments of Gastroenterology and Neurology, Faculty of Medicine, Kagawa University, 1750-1, Ikenobe, Miki-cho, Kita-gun, Kagawa 761-0793 Japan; 3 Radiation Oncology, Faculty of Medicine, Kagawa University, 1750-1, Ikenobe, Miki-cho, Kita-gun, Kagawa 761-0793 Japan

**Keywords:** Inflammatory nutritional factor, PDAC, NACRT

## Abstract

**Background:**

Inflammatory nutritional factors, such as the neutrophil/lymphocyte ratio (NLR), Glasgow Prognostic Score (GPS), modified GPS (mGPS), and C-reactive protein/albumin (CRP/Alb) ratio, have prognostic values in many types of cancer. In this study, the prognostic values of inflammatory nutritional scores were evaluated in the patients with resectable or borderline resectable pancreatic ductal adenocarcinoma (PDAC) after neoadjuvant chemoradiotherapy (NACRT).

**Methods:**

A total of 49 patients who underwent pancreatectomy after NACRT from September 2009 to May 2016 were enrolled. The NACRT consisted of hypofractionated external-beam radiotherapy (30 Gy in 10 fractions) with concurrent S-1 (60 mg/m^2^) delivered 5 days/week for 2 weeks before pancreatectomy. Inflammatory nutritional scores were determined before and after NACRT in this series.

**Results:**

The median NLR increased after NACRT (from 2.067 to 3.302), with statistical difference (*p* < 0.001). In multivariate analysis, high pre-NACRT mGPS (2 or 1; *p* = 0.0478) and significant increase in CRP/Alb ratio after NACRT (≧ 0.077; *p* = 0.0036) were associated with shorter overall survival. All patients were divided into two groups according to the ΔCRP/Alb ratio after NACRT: the group with high ΔCRP/Alb ratio (≧ 0.077) and the group with low ΔCRP/Alb ratio (< 0.077). The group with high ΔCRP/Alb ratio after NACRT (n = 13) not only had higher post-NACRT CRP levels (*p* < 0.001) but also had lower post-NACRT Alb levels (*p* = 0.002). Patients in the group with high ΔCRP/Alb ratio lost more body weight during NACRT (*p* = 0.03).

**Conclusion:**

In addition to pre-NACRT mGPS, ΔCRP/Alb after NACRT could provide prognostic value in the patients with PDAC treated by NACRT.

## Background

Pancreatic ductal adenocarcinoma (PDAC), as a representative lethal malignant disease, is the fourth leading cause of cancer death worldwide and has the worst prognosis, with less than 8% of patients surviving 5 years after diagnosis [1]. Although surgical resection provides only chance for cure, the resection rate is very low (15–20%) [2]. Additionally, even after surgical resection with curative pathological findings, an estimated 80–85% of patients experience recurrence, with a median survival of 20–24 months [3–5]. The therapeutic agent added to surgery, particularly adjuvant chemotherapy, appears to be necessary and effective [2, 6]. Advanced research in adjuvant and neoadjuvant therapies, including chemotherapy, radiotherapy, and chemoradiotherapy, has focused on its therapeutic effect to PDAC [7–10].

Determining the prognostic factors is of importance in facilitating adequate treatment for patients with PDAC and identifying patients who might have poor prognosis and would not benefit from the treatment. Some studies have described various prognostic predicting factors for patients with resectable (R) or borderline resectable (BR) PDAC, e.g., age, tumor size, tumor marker carbohydrate antigen 19-9 level, resection margins, lymph node metastasis, and postoperative complications [11–14].

Recently, inflammation-based prognostic scores and nutritional status, such as the neutrophil/lymphocyte ratio (NLR), C-reactive protein/albumin (CRP [mg/L]/Alb [g/L]), Glasgow Prognostic Score (GPS), and modified GPS (mGPS), are considered to be associated with tumor progression and prognosis in various carcinomas [15–19]. Some research indicated that inflammatory nutritional scores are associated with the prognosis of the patients with PDAC [15, 20, 21]. However, few reports have described the values of such prognostic scores focusing on the patients treated by neoadjuvant chemoradiotherapy (NACRT), including those with other types of cancers [22].

NACRT was introduced for R and BR—PDAC in 2009 with approval from the ethical committee in our institution [23]. In the current study, the prognostic values of inflammatory nutritional scores were evaluated in the patients who had been enrolled in this Phase II clinical trial.

## Methods

### NACRT

The previous NACRT study was a single-center, prospective phase II trial in the patients who had histologically diagnosed PDAC (UMIN-CTR #UMIN000026438). Hypofractionated external-beam radiation (30 Gy in 10 fractions) with concurrent S-1 (60 mg/m2/day) was delivered 5 days per week for 2 weeks. After restaging, pancreatic resection was performed appropriately 4 weeks after the initiation of the protocol treatment. Eligibility criteria of this NACRT protocol included patients with R and BR PDAC (based on the NCCN guideline) [24], performance status 0–1, age 20–85 years, adequate organ function (defined by hemoglobin ≥ 9 g/dL, absolute neutrophil count ≥ 2 × 10^9^/L, platelet count ≥ 100 × 10^9^/L, total bilirubin ≤ 6.0 mg/dL, serum transaminases ≤ 3 times the upper normal limit, and creatinine clearance ≥ 60 mL/min [23]. All patients provided written informed consent before inclusion in the study, which was approved by the institutional review board of Kagawa University. This phase II trial enrolled 57 patients from September 2009 to May 2016.

### Patient selection

**Fig. 1:**
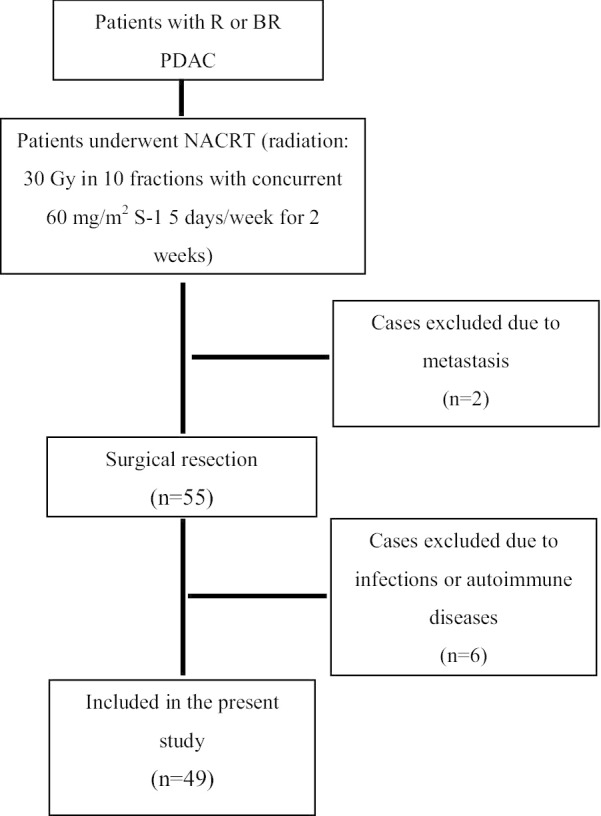
Patient selection and treatment chart. R, resectable; BR, borderline resectable; PDAC, pancreatic ductal adenocarcinoma; NACRT, neoadjuvant chemoradiotherapy

Fifty-five patients eventually underwent pancreatic resection after the NACRT. Excluding 6 patients who were complicated with infection diseases, mostly acute cholangitis, and had autoimmune diseases required medication before or during NACRT, a total of 49 patients were included the present study (Fig. 1).

### Surgery

Pancreaticoduodenectomy (PD) with regional lymphadenectomy was performed according to the Whipple procedure. The reconstruction after PD was performed with the modified Child’s method. Distal pancreatectomy (DP) was performed basically with radical antegrade modular pancreatosplenectomy (RAMPS) procedures.

### Definition of inflammatory nutritional prognostic scores

The inflammatory nutritional prognostic scores were determined before NACRT and after NACRT (before surgical resection) in this study. CRP/Alb meant CRP (mg/L)-albumin (g/L) ratio. GPS and mGPS were calculated in accordance with original papers as descried in Additional file 1: Table S1 [18, 19].

### Statistical analyses

The survival curve was estimated using the Kaplan–Meier method. Overall survival (OS) and disease-free survival (DFS) were defined as the interval from the start of NACRT to death from any cause and recurrence or to the last follow-up. Statistical data were obtained using the BellCurve for Excel (Social Survey Research Information Co., Ltd.). The 75% level of the continuous variables was defined as the cutoff for the statistical analyses.

### Comparison based on inflammatory nutritional scores

Considering the results of the prognostic scores, the patients in the present study were divided into two groups based on the inflammatory nutritional scores. Additional assessment was performed to determine the factors affecting the inflammatory nutritional scores associated with poor PDAC prognosis.

## Results

Table 1 summarized the patient characteristics. The tumor was located in the proximal pancreas in 36 patients and in the distal pancreas in 13. Forty-one patients with R PDAC (84%) and 8 with BR PDAC (16%) were included. Seven patients had BR with involvement of the portal vein (BR-PV) and 1 had BR with arterial abutment (BR-A). R0 resection was achieved in 35 of the 49 patients (71.4%). Pathological tumor response was classified as Evans grade I in 3 patients, IIa in 30 patients, IIb in 14 patients, III in 1 patient, and IV in 1 patient. The postoperative morbidity (Clavien-Dindo grade IIIb or more) was observed in 12 (25%) patients. Twenty-seven (55%) patients completed adjuvant chemotherapy with S-1 or gemcitabine for 6 months according to the recommended protocols. The median observation period of this study was 33 months. In the present series, the 1-, 3-, and 5-year OS rates with the Kaplan–Meier survival estimates were 89.6%, 52.5%, and 39.4%, respectively.

**Table 1:** Patient characteristics (n = 49)

Age (years)	70 (61–82)
Tumor size (mm)	22 (10–70)
Sex	
Male	27 (55.1)
Female	22 (44.9)
Primary tumor location	
Head/neck	36 (73.5)
Body/tail	13 (26.5)
Operation procedure	
Pancreatoduodenectomy	34 (69.4)
Distal Pancreatectomy	11 (22.4)
Total Pancreatectomy	4 (8.2)
NCCN resectability (2015)	
Resectable	41 (83.7)
Borderline resectable	
BR-PV^a^	7 (14.3)
BR-A^a^	1 (2)
Operation time (min)	477 (231–816)
Intraoperative bleeding (mL)	1360 (231–9268)
Postoperative hospital stays (days)	25 (14–152)
Complication after surgery	12 (24.5)
Uncompleted adjuvant chemotherapy	22 (44.9)

Data are presented as median (range) or n (%). Each value was assessed before neoadjuvant chemoradiotherapy

NCCN, National Comprehensive Cancer Network; BR-PV, borderline resectable with portal vein invasion; BR-A, borderline resectable with arterial invasion

^a^According to NCCN guideline 2018 [1]

**Fig. 2:**
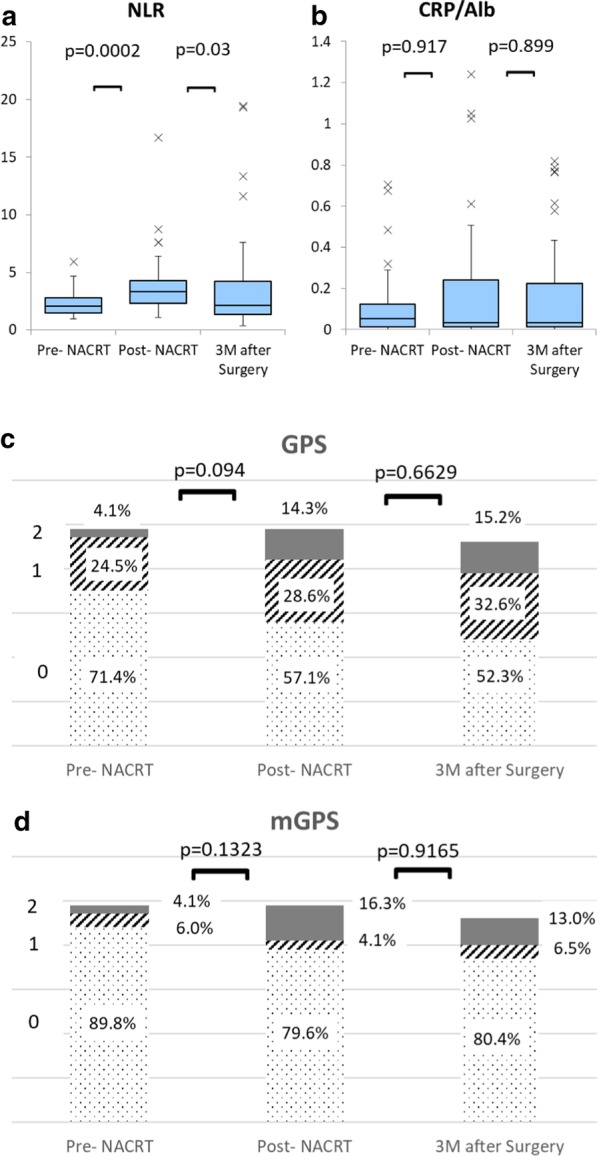
Change in inflammatory nutritional scores during neoadjuvant chemoradiotherapy and after surgery. NLR, neutrophil/lymphocyte ratio; GPS, Glasgow Prognostic Score; mGPS, modified Glasgow Prognostic Score; CRP/Alb, C-reactive protein/albumin ratio

Changes of inflammatory nutritional scores before NACRT (pre NACRT), after NACRT (post NACRT) (equal to immediately before surgery) and 3 months after surgery are shown in Fig. 2. NLR increased after NACRT (from 2.067 to 3.302) and decreased 3 months after surgery (from 3.302 to 2.148, *p* = 0.03) with statistically significant differences (*p* < 0.001 and *p* = 0.03, respectively) (Fig. 2a). In contrast, GPS, mGPS and CRP/Alb did not change significantly between NACRT and at 3 months after surgery (Fig. 2b–d).

According to the statistical analysis, the 75% level of the continuous variable was defined as the cutoff (Table 2). In the univariate Cox regression analysis, resectability (borderline resectable; *p* = 0.003), high pre-NACRT mGPS (2 or 1; *p* = 0.007), high post-NACRT CRP/Alb ratio (≧ 0.241; *p* = 0.0012), and significant increase in CRP/Alb ratio (high ΔCRP/Alb) after NACRT (≧ 0.077; *p* = 0.006) were significantly associated with shorter OS. Tumor size in post-NACRT was not included in the regression analysis because it is difficult to measure tumor size accurately due to effect of radiation.

**Table 2:** Analysis of the association between clinical characteristics and OS

	Cutoff value	Univariate analysis		Multivariate analysis	
		Hazard ratio	*p* value	Hazard ratio	*p* value
Age (years)	≧ 70	1.1455	0.7395		
Sex	Female	1.4304	0.3828		
Location	Head	0.8938	0.8142		
Resectability	BR-A or BR-PV	*3.9383*	*0.0033*	0.9127	0.8896
<Pre-NACRT>					
Tumor size (mm)	≧ 20	1.6170	0.3436		
CEA (ng/mL)	≧ 5.8	0.9582	0.9289		
CA19-9 (U/mL)	≧ 993	1.6661	0.2444		
FDG-PET (SUVmax)	≧ 10.87	0.9028	0.8294		
NLR	≧ 2.79	2.2490	0.0583		
Alb (g/dL)	≦ 3.5	0.9777	0.9624		
GPS	2 or 1	1.3676	0.4946		
mGPS	2 or 1	*4.7085*	*0.007*	*3.8635*	*0.0478*
CRP/Alb ratio	≧ 0.124	2.2201	0.0738		
<Post-NACRT>					
CEA (ng/mL)	≧ 6.5	1.0509	0.1986		
CA19-9 (U/mL)	≧ 483	1.1494	0.7716		
NLR	≧ 4.30	1.9694	0.1229		
Alb (g/dL)	≦ 3.2	2.1969	0.0665		
GPS	2 or 1	1.9590	0.1087		
mGPS	2 or 1	2.3647	0.0603		
CRP/Alb ratio	≧ 0.24	*2.9130*	*0.00122*		
<Change of inflammatory response factor after NACRT>					
Δ: Post-NACRT-pre-NACRT					
ΔNLR	1.79	0.9952	0.9971		
GPS	Increase	2.2562	0.0583		
mGPS	Increase	2.3647	0.0603		
ΔCRP/Alb ratio	0.0769	*3.2706*	*0.0060*	*5.1842*	*0.0036*
<Perioperative factor>					
Operative time (min)	≧ 560	1.8544	0.1999		
Intraoperative bleeding (mL)	≧ 2088	*2.4717*	*0.0387*	1.1879	0.7548
Complication after surgery	≧ IIIa	0.6719	0.4338		
Postoperative hospital stays (days)	≧ 43	0.7421	0.5302		
Adjuvant therapy	In-completion	1.9714	0.0990		
<Pathological factor>					
Tumor size	≧ 20	2.8865	0.1536		
T	T3	1.4130	0.5319		
Differentiation	Moderate or poor	1.6448	0.2581		
Lymph node metastasis	N2 or N1	*3.5747*	*0.0080*	*4.7976*	*0.0117*
ly	2 or 3	1.3131	0.59647		
v	2 or 3	1.7984	0.1657		
ne	2 or 3	1.4089	0.4079		
PV	1	*5.6948*	*0.0001*	2.6003	0.1012
Surgical margins	R1	*12.1127*	*0.0042*	*12.6514*	*0.0295*
Evans	I or IIa	2.1983	0.1255		

BR-A, borderline resectable with arterial invasion; BR-PV, borderline resectable with portal vein invasion; NACRT, neoadjuvant chemoradiotherapy; CEA, carcinoembryonic antigen; CA19-9, carbohydrate antigen 19-9; FDG-PET, fluorodeoxyglucose positron emission tomography; SUVmax, maximum standardized uptake value; NLR, neutrophil/lymphocyte ratio; Alb, albumin; GPS, Glasgow Prognostic Score; mGPS, modified Glasgow Prognostic Score; CRP, C-reactive protein; PV, portal vein

According to the perioperative course and pathological findings, intraoperative bleeding (≧ 2088 mL; *p* = 0.04), lymph node metastasis (N1 or N2; *p* = 0.008), portal vein invasion (PVI; 0.0001), and positive surgical margin (R1; *p* = 0.004) were significantly associated with shorter OS.

In the multivariate survival analysis using a Cox proportional hazards model, high pre-NACRT mGPS (2 or 1; *p* = 0.0478), significant increase in CRP/Alb ratio (high ΔCRP/Alb) after NACRT (≧ 0.077; *p* = 0.0036), lymph node metastasis (N1 or N2; *p* = 0.0117), and positive surgical margin (R1; *p* = 0.0295) were associated with shorter OS, with statistical differences (Table 2). The prognostic value of high post-NACRT CRP/Alb ratio was excluded in the multivariate analysis because high post-NACRT CRP/Alb ratio was closely related with a significant increase in CRP/Alb ratio after NACRT.

**Fig. 3:**
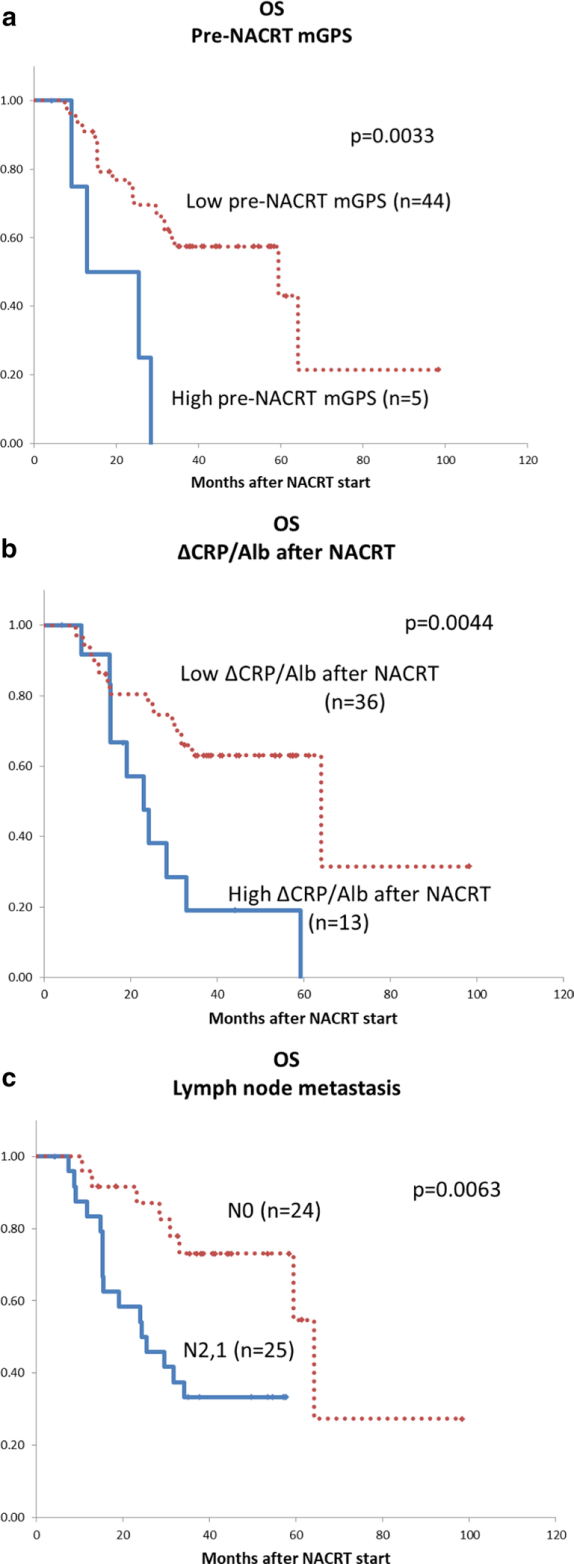
Overall survival estimated with the Kaplan–Meier method. **a** Pre-NACRT mGPS, **b** ΔCRP/Alb ratio after NACRT, **c** lymph node metastasis. mGPS, modified Glasgow Prognostic Score; NACRT, neoadjuvant chemoradiotherapy; CRP/Alb, C-reactive protein/albumin ratio

The Kaplan–Meier survival estimates showed poor OS, with median OS of 12.8 months in the group with high pre-NACRT mGPS (2 or 1) compared with 59.3 months in the group with low pre-NACRTmGPS (0) (*p* = 0.003) (Fig. 3a). In addition, median OS were 23.1 and 64.1 months in the high (≧ 0.077) and low (< 0.077)Δ CRP/Alb groups (*p* = 0.004) (Fig. 3b). The N2 or N1 group also demonstrated poor OS, with statistical differences (*p* = 0.006) (Fig. 3c).

**Fig. 4:**
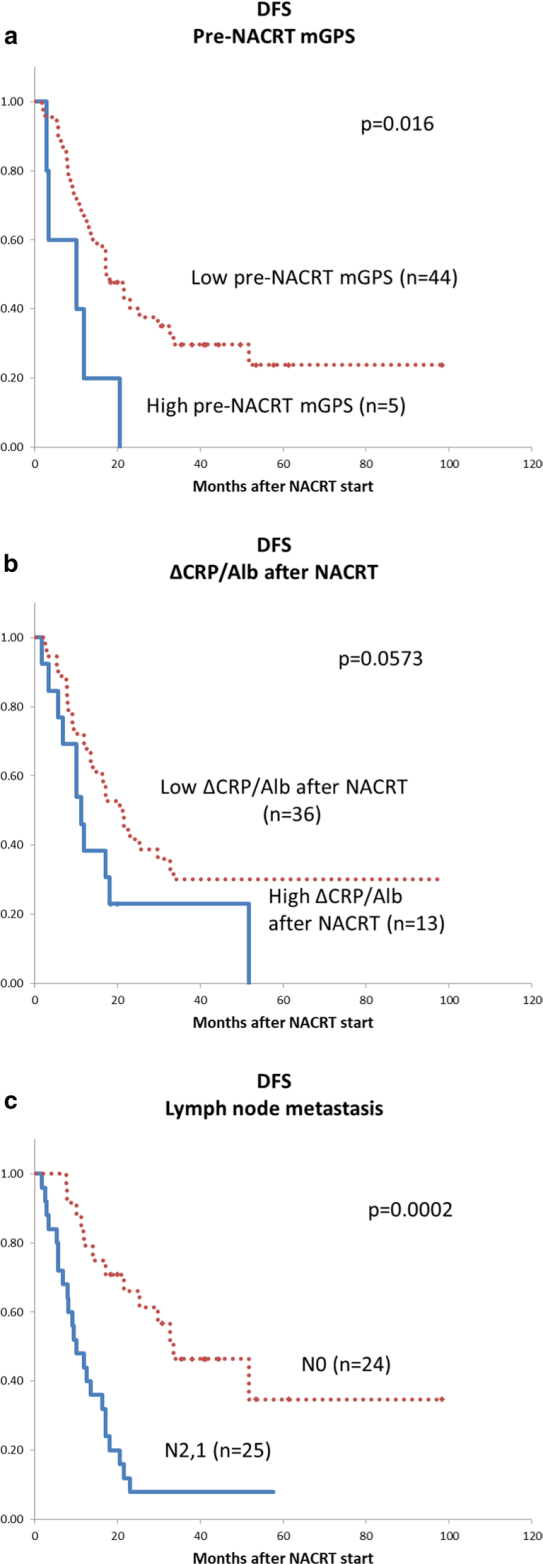
Disease-free survival estimated with the Kaplan–Meier method. **a** Pre-NACRT mGPS, **b** ΔCRP/Alb ratio after NACRT, **c** lymph node metastasis. mGPS, modified Glasgow Prognostic Score; NACRT, neoadjuvant chemoradiotherapy, CRP, C-reactive protein; Alb, albumin

DFS were analyzed with the Kaplan–Meier log-rank test. DFS in the group with high pre-NACRT mGPS (2 or 1) and the N1 group were poor, with statistical differences (*p* = 0.016, *p* = 0.0002) (Fig. 4a, c). Although the p-value did not reach statistical difference, DFS of the group with high ΔCRP/Alb was shorter compared with the group with low Δ CRP/Alb (*p* = 0.0573) (Fig. 4b).

All the patients included this study were divided into two groups according to ΔCRP/Alb ratio after NACRT: the group with high ΔCRP/Alb ratio (≧ 0.077) and the group with low ΔCRP/Alb ratio (< 0.077) (Table 3). The group with high ΔCRP/Alb ratio after NACRT (n = 13) not only had higher post-NACRT CRP levels (*p* < 0.001) but also had lower post-NACRT Alb levels (*p* = 0.0015) than the group with low ΔCRP/Alb ratio. The patients with high ΔCRP/Alb ratio lost more body weight after NACRT (*p* = 0.03). There was no significant difference in tumor-related factors (e.g., tumor size, tumor marker, or lymph node metastasis) between the high and low ΔCRP/Alb groups.

**Table 3:** Characteristics of patients in the groups with high ΔCRP/Alb ratio (≧ 0.077) and the group with low Δ CRP/Alb ratio (< 0.077)

	High Δ CRP/Alb group (n = 13)	Low Δ CRP/Alb group (n = 36)	*p* value
Age (years)	74	68	0.1663
Sex: female	6 (46.2)	16 (44.4)	0.916
Tumor size (mm)	30	20	0.076
<Pre-NACRT>			
CEA (ng/mL)	5.5	3.9	0.1235
CA19-9 (U/mL)	537	214	0.1889
FDG-PET (SUVmax)	7.8	7.0	0.5926
Resectability: BR	2 (15.4)	6 (16.7)	0.9155
BMI	23.5	22.1	0.1129
CRP (mg/dL)	0.41	0.16	0.0698
Alb (g/dL)	3.8	3.9	0.7941
NLR	2.51	2.00	0.0852
GPS 2 or 1	4 (30.8)	10 (27.8)	0.8395
mGPS 2 or 1	2 (15.4)	3 (8.3)	0.4761
<Post-NACRT>			
CEA (ng/mL)	6.5	3.5	0.1333
CA19-9 (U/mL)	455	107	0.0728
* ΔWeight loss (kg)*	*1.55*	*0.48*	*0.0314*
* CRP (mg/dL)*	*1.32*	*0.08*	*< 0.0001*
* Alb (g/dL)*	*3.2*	*3.9*	*0.0015*
NLR	3.20	3.34	0.9639
* GPS 2 or 1*	*11 (84.6)*	*10 (27.8)*	*0.0004*
* mGPS 2 or 1*	*10 (76.9)*	*0 (0)*	*< 0.0001*
<Perioperative and postoperative factor>			
Intraoperative bleeding (mL)	1764	1005	0.0666
Complication after surgery^a^	2 (15.4)	10 (27.8)	0.3780
Postoperative hospital stays (days)	29	25	0.5183
T3	2 (15.4)	5 (13.9)	0.8960
N2 or N1	7 (53.8)	18 (50.0)	0.8139
PVI	5 (38.5)	10 (27.8)	0.4783
Surgical margin: R1	4 (30.8)	10 (27.8)	0.8395
Evans I or IIa	11 (84.6)	22 (61.1)	0.1252
Uncompleted adjuvant chemotherapy	7 (53.9)	15 (41.7)	0.4539

Data are median or n (%)

CRP, C-reactive protein; Alb, albumin; NACRT, neoadjuvant chemoradiotherapy; CEA, carcinoembryonic antigen; CA19-9, carbohydrate antigen 19-9; FDG-PET, fluorodeoxyglucose positron emission tomography; SUVmax, maximum standardized uptake value; borderline resectable; BMI, body mass index; NLR, neutrophil/lymphocyte ratio; GPS, Glasgow Prognostic Score; mGPS, modified Glasgow Prognostic Score; PVI, portal vein invasion

^a^Complication after surgery: Clavien-Dindo ≧ IIIa

## Discussion

The prognosis of PDAC depends on biological behavior and patient-related factors [2, 25]. Some factors are known predictive prognosis factors of PDAC, such as age, tumor size, pathological type, lymph node metastasis, and vascular invasion [2]. Recently, some papers described patient-related factors that affect the prognosis of some cancers that are as useful as common prognosis factors. Inflammation- and nutrition-based prognostic scores, such as the NLR, GPS, mGPS, and CRP/Alb ratio have been reported to have prognostic values in many types of cancer [15–19]. Chronic inflammation has a direct causal relationship with tumorigenesis, and malignancies trigger an inflammatory response that leads to deleterious effects on the malignant process [26]. Cancer-related inflammation causes suppression of antitumor immunity through recruitment of regulatory T cells and activation of chemokines that encourage tumor growth and metastasis [27]. Patients with PDAC are usually malnourished and thus immunocompromised [28]. Impaired host immunity can contribute to tumor growth [29]. Some studies of inflammatory response–related scoring systems in assessing the prognosis of patients with PDAC concluded that NLR, GPS, mGPS, and CRP/Alb ratio may be useful for predicting prognosis [15, 20, 21].

Although PDAC remains a highly lethal disease, neoadjuvant therapy has been considered to provide a new and effective treatment strategy for PDAC. It improves treatment outcome in resectable and borderline resectable cases and provides a survival benefit in patients with advanced stages of PDAC [5, 7, 30]. Because neoadjuvant therapy for resectable or borderline resectable PDAC is not yet a standard treatment, NACRT has been the subject of clinical trials at high-volume centers in Japan. Because of this, there are few studies that demonstrate the value of inflammatory nutritional prognostic scores in the patients undergoing NACRT for resectable or borderline resectable PDAC. This is the first report showing that increased CRP/Alb ratio after NACRT can be a valuable prognostic factor. Our previous study presented that neoadjuvant S-1 with concurrent hypofractionated radiotherapy is tolerable and appears promising for patients with resectable and borderline resectable PDAC [23]. Recently, a randomized phase II/III trial of neoadjuvant chemotherapy with gemcitabine and S-1 versus upfront surgery for resectable pancreatic cancer (Prep-02/JSAP05) (clinical trial information: UMIN000009634) demonstrated that neoadjuvant chemotherapy contributed to improved treatment outcome and prognosis for resectable PDAC compared with upfront surgery [31]. Based on the efficacy of neoadjuvant chemotherapy in the planned resection of patients with PDAC, as determined by the trial, neoadjuvant therapy could become one of the standard established therapies for resectable or borderline resectable PDAC.

This study demonstrates that high pre-NACRT mGPS and increased CRP/Alb ratio after NACRT are independent prognostic factors for patients with resectable or borderline resectable PDAC undergoing NACRT following pancreatic resection, like lymph node metastasis and cancer remnant. Both OS and DFS estimated with the Kaplan–Meier method are poor in the patient group with high pre-NACRT mGPS. According to the patient group with increased CRP/Alb ratio after NACRT, OS was poor. Additionally, DFS was also inferior although the *p* value did not reach statistical difference. Several papers have demonstrated that some preoperative inflammatory nutritional scores could be utilized as prognosis predictors [15, 20, 21]. Additionally, a study reported that an increase in NLR postoperative course leads to poor prognosis [32]. Furthermore, from the results of this study, a change in the degree of inflammatory nutritional scores after NACRT could also be valuable predictive markers. To our knowledge, there are only a few reports that focused on the relationship between NACRT for PDAC and change in inflammatory nutritional scores. Differences in race may affect the findings in the present study, as S-1 is used as sensitizer in the protocol. Further examinations focusing on changes in inflammatory nutritional scores are required, because treatment protocols for NACRT differ among institutions.

To assess the cause of increased CRP/Alb ratio after NACRT, all the patients included in this study were divided into two groups according to ΔCRP/Alb ratio after NACRT: the group with high ΔCRP/Alb (≧ 0.077) and the group with low-ΔCRP/Alb ratio (< 0.077). The group with high ΔCRP/Alb after NACRT not only had higher post-NACRT CRP level but also lower post-NACRT Alb level and lost more body weight after NACRT than the group with low-ΔCRP/Alb. Previous studies reported that tumor malignant behavior and progression are associated with impaired Alb synthesis and malnutrition. However, tumor characteristics, including tumor size, tumor marker and lymph node metastasis, were not significantly different between the two groups, suggesting that low Alb levels and malnutrition are independently related to poor prognosis. Meanwhile, some papers have described sarcopenia or loss of skeletal muscle volume as adverse prognostic factors for pancreatic cancer [33–35]. Based on the reason for the high post-NACRT CRP level in the group with high − ΔCRP/Alb, the patients could be more prone to development of inflammation due to carcinogenesis and/or NACRT.

Some reported that predictive prognostic factors in PDAC, including tumor size, tumor marker, lymph node metastasis, differentiation, and molecular behavior, could not be intervened enough for the improvement of prognosis. Tumor behavior and pathological examinations are difficult to access before surgical resection. Meanwhile, assessment of immune nutritional status through CRP/Alb ratio and mGPS are available from the first diagnosis up to the postoperative period. Most important, malnutrition and sarcopenia can be modified with appropriate intervention. Patients who undergo NACRT, especially those with low albumin level or with low body weight, should be given advance nutritional support because they have enough time to improve their albumin level and/or body weight before surgery.

Several limitations of the study should be mentioned. First, the number of patients in the study may not be sufficient to draw firm conclusions based on statistical analysis. Since the use of NACRT has not been standardized widely to date, collecting enough patients treated using the same protocols is currently difficult. The second limitation is that the inflammation and nutritional scores may be affected by progression of TNM stage due to the heterogeneous patient population in the study. We hope that the proposed consequences of the study will be verified by future large-scale research with subgroup analysis of patients at an identical stage.

## Conclusion

Inflammatory nutritional prognostic scores could provide additional prognostic value even in patients with PDAC treated by NACRT. Further studies are needed in order to consider the possibility that advanced nutrition support during NACRT might be able to contribute to improving the prognosis.

## Supplementary Information

**Additional file 1: Table 1.** Inflammatory nutritional prognostic scoring system.

## Data Availability

The datasets generated and/or analyzed in the present study are available from the corresponding author on reasonable request.

## Abbreviations

PDAC: Pancreatic ductal adenocarcinoma
NACRT: Neoadjuvant chemoradiotherapy
NLR: Neutrophil/lymphocyte ratio
GPS: Glasgow prognostic score
mGPS: Modified Glasgow prognostic score
CRP/Alb ratio: C-reactive protein/albumin ratio
R: Resectable
BR: Borderline resectable
NCCN: The National Comprehensive Cancer Network
PD: Pancreaticoduodenectomy
DP: Distal pancreatectomy
RAMPS: Radical antegrade modular pancreatosplenectomy
OS: Overall survival
DFS: Disease-free-survival
BR-PV: Borderline resectable with portal vein invasion
BR-A: Borderline resectable with arterial invasion
CEA: Carcinoembryonic antigen
CA19-9: Carbohydrate antigen 19-9
FDG-PET: Fluorodeoxyglucose positron emission tomography
SUV max: Maximum standardized uptake value
BMI: Body mass index
PVI: Portal vein invasion
