# Supplementary material for: Changes and prognostic impact of inflammatory nutritional factors during neoadjuvant chemoradiotherapy for patients with resectable and borderline resectable pancreatic cancer
Source: BMC Gastroenterol. 2020 Dec 14;20:423. doi: 10.1186/s12876-020-01566-8 (PMC7734830; doi:10.1186/s12876-020-01566-8)
Supplement: Supplementary file 1 — Additional file 1: Table 1. Inflammatory nutritional prognostic scoring system. [file 12876_2020_1566_MOESM1_ESM.docx]

Additional Table 1. Inflammatory nutritional prognostic scoring system

| Glasgow Prognostic Score |  |
| --- | --- |
| CRP ≦1.0 mg/L/albumin ≧3.5 g/dL | 0 |
| CRP ≦1.0 mg/L/albumin <3.5 g/dL | 1 |
| CRP >1.0 mg/L/ albumin ≧3.5 g/dL | 1 |
| CRP >1.0 mg/L/albumin <3.5 g/dL | 2 |
| Modified Glasgow Prognostic Score |  |
| CRP ≦1.0 mg/L/albumin ≧3.5 g/dL | 0 |
| CRP ≦1.0 mg/L/albumin <3.5 g/dL | 0 |
| CRP >1.0 mg/L/albumin ≧3.5 g/dL | 1 |
| CRP >1.0 mg/L/albumin <3.5 g/dL | 2 |

CRP, C-reactive protein; Alb, albumin.
